# Applicability of polygenic risk scores in endometriosis clinical presentation

**DOI:** 10.1186/s12905-022-01788-w

**Published:** 2022-06-03

**Authors:** Agnes Svensson, Koldo Garcia-Etxebarria, Anna Åkesson, Christer Borgfeldt, Bodil Roth, Malin Ek, Mauro D’Amato, Bodil Ohlsson

**Affiliations:** 1 Department of Internal Medicine, Skåne University Hospital, Lund University, Malmö, Sweden; 2 Biodonostia, Gastrointestinal Genetics Group, Centro de Investigación Biomédica en Red de Enfermedades Hepáticas Y Digestivas (CIBERehd), 20014 San Sebastian, Spain; 3 Clinical Studies Sweden – Forum South, Skåne University Hospital, Lund, Sweden; 4 Department of Obstetrics and Gynecology, Skåne University Hospital, Lund University, Lund, Sweden; 5 Gastrointestinal Genetics Lab, CIC bioGUNE - BRTA, Derio, Spain; 6 Ikerbasque, Basque Foundation for Science, Bilbao, Spain; 7 Department of Medicine and Surgery, LUM University, Casamassima, Italy

**Keywords:** Endometriosis, Gastrointestinal symptoms, Genetics, Inflammatory proteins, Polygenic risk score

## Abstract

**Background:**

Risk prediction is an essential part of preventative medicine and in recent years genomic information has become an interesting factor in risk models. Polygenic risk scores (PRS) combine the effect of many genetic variations into a single score which has been shown to have predictive value for many diseases. This study aimed to investigate the association between PRS for endometriosis and the clinical presentation of the disease.

**Methods:**

Women with endometriosis (N = 172) were identified at the Department of Gynecology. All participants answered questionnaires regarding sociodemographic factors, lifestyle habits and medical history, registered bowel symptoms on the Visual Analog Scale for Irritable Bowel Syndrome and passed blood samples. DNA was extracted and samples were genotyped, and a PRS was calculated based on previous genome-wide association studies of endometriosis. Inflammatory proteins and TSH receptor antibodies (TRAb) in serum were analyzed.

**Results:**

Inverse associations were identified between PRS and spread of endometriosis, involvement of the gastrointestinal tract and hormone treatment. However, significance was lost when calculated as p for trend and the specificity and sensitivity were low. There were no correlations between PRS and TRAb or inflammatory proteins.

**Conclusion:**

The findings indicate that specific PRS should be developed to predict clinical presentations in patient with endometriosis.

**Supplementary Information:**

The online version contains supplementary material available at 10.1186/s12905-022-01788-w.

## Background

Endometriosis is a chronic, inflammatory gynecological disease, affecting up to 10% of women of reproductive age [1, 2]. The disease is defined by ectopic endometrial cells and stroma growing outside of the uterus and is associated with dysmenorrhea, pelvic pain, gastrointestinal symptoms and infertility. The most established theory of pathogenesis is Sampson´s theory of retrograde menstruation [3]. However, retrograde menstruation has been seen in up to 90% of women [4], which indicates that other mechanisms, such as genetic, immunological and environmental factors are involved in the development of endometriosis [5]. New theories also suggest that bone marrow-derived stem cells might migrate through the peripheral circulation, causing endometriosis in sites outside of the peritoneal cavity [6].

The hereditary component of endometriosis, explained by genetic variations, has in previous studies been estimated to 47–51% [7, 8]. There are different types of genetic variations contributing to disease. One common form of allelic variation is Single Nucleotide Polymorphism (SNP) and in total, SNPs explain approximately 26% of the polygenic risk of endometriosis [9]. Genome-wide association studies (GWAS) are commonly used to discover genetic variants associated with a disease [10, 11]. Several GWAS of endometriosis have identified genetic variants related to development and regulation of the female reproductive tract [12]. Individually, these genetic variants have low impact on disease risk. Calculating a polygenic risk score (PRS) is a way to assess the risk of a condition based on the combined effect of many genetic variants. Using a PRS has in many diseases been shown to be better for risk prediction than individual SNPs [13, 14]. However, to our knowledge, PRS has never been applied for endometriosis.

Endometriosis is widely known as an inflammatory condition and serum concentrations of the inflammatory proteins Axis inhibition protein 1 (AXIN1), Sulfotransferase Family 1A Member 1 (ST1A1) and C-X-C Motif Chemokine Ligand 9 (CXCL9) have been found to be altered in women with endometriosis [15]. The inflammatory proteins Oncostatin-M (OSM), Monocyte Chemoattractant protein 1 (MCP-1) and TNF Receptor Superfamily Member 9 (TNFRSF9) have been shown to be associated with different symptoms of endometriosis [15]. Several studies have highlighted the association between thyroid dysfunction and endometriosis [16–18]. Recently, elevated titers of thyroid-stimulating hormone (TSH) receptor antibodies (TRAb) have been shown to be associated with endometriosis, suggesting a connection between the two pathologies [19].

The primary aim of the present study was to investigate the association between PRS for endometriosis and clinical presentations of the disease. Secondary aims were to investigate associations of PRS for different inflammatory proteins and TRAb.

## Materials and methods

Ethical consent for the study was obtained from the Ethics Review Board of Lund University, No 2012/564, 2016/56.

### Study participants and design

Women with endometriosis were identified at the Department of Gynecology at Skåne University Hospital, Malmö, according to the ICD-10 classification of endometriosis, N80. The recruitment took place between March 2013 and March 2017 according to previously described inclusion and exclusion criterium [17]. The main inclusion criterium was to have a definite diagnosis of endometriosis confirmed by laparoscopy or laparotomy. General inclusion criteria were to comprehend the Swedish or English language and an age above 18 years. Exclusion criteria were an uncertain diagnosis of endometriosis, multiple or severe somatic or psychiatric comorbidities, a diagnosis of inflammatory bowel disease (IBD), current pregnancy or living too far from the hospital. In total, 172 women were finally included in the study.

All study participants answered a questionnaire and completed the Visual Analog Scale for Irritable Bowel Syndrome (VAS-IBS). They also passed blood samples, and whole blood and sera were kept frozen at − 80 °C. Inflammatory proteins and TRAb in serum were analyzed. DNA was extracted and samples were genotyped, and a PRS was calculated based on previous GWAS of endometriosis.

### Study questionnaire

A previously developed questionnaire regarding socioeconomic factors, medical history and pharmacological treatments was completed by all study participants. In the questionnaire they also answered questions about their endometriosis-associated symptoms such as onset of the disease, trigger factors, examinations and treatments (Additional file 1) [19].

### The visual analog scale for irritable bowel syndrome

A validated, unlicensed questionnaire called VAS-IBS was used to estimate bowel symptoms (Additional file 2) [20]. The patients used the VAS-IBS questionnaire to estimate the severity of their symptoms over the past 2 weeks regarding abdominal pain; constipation; diarrhea; bloating and flatulence; vomiting and nausea; and intestinal symptom’s influence on daily life, as previously described [17]. They estimated their symptoms on a continuous scale from 0 to 100 mm where 0 represents no symptoms and 100 represents severe symptoms. The scales were inverted from the original format [20]. Reference values are based on controls, recruited among hospital staff at Skåne University Hospital, Malmö, consisting of 52 women who had not undergone prior abdominal surgery, with a median age of 44 (range 22–77) years [21].

### Biological sample analyses

A multiplex proximity extension assay, Proseek^®^Multiplex Inflammation 1 kit (Olink Bioscience, Uppsala, Sweden), was used to analyze 92 inflammatory proteins in serum, as previously described [15]. Measured proteins are expressed in an arbitrary unit on a log2 scale celled Normalized Protein Expression (NPX). Larger NPX values represent higher protein expressions in the sample. The proteins ST1A1, CXL9, OSM, MCP-1 and TNFRSF9 from this analysis, who had previously been shown to be associated with endometriosis and gastrointestinal symptoms, were used in this study [13]. Sandwich ELISA technology (MBS762601, MyBiosource, San Diego, CA, USA, lot.no.H2497C119) was used to analyze AXIN1 in plasma, as previously described [13]. Analysis of TRAb IgG autoantibodies was conducted in serum according to clinical routines, using a competitive Electro Chemi Luminescence Immunoassay (ECLI) detection technique based on Ruthenium deriva [22].

### Sample sequencing and quality control

DNA samples of 172 patients were genotyped using Illumina Global Screening Array, on Illumina iScan high-throughput screening system at the Institute of Clinical Molecular Biology (Christian-Albrechts-University, Kiel, Germany). The GenCall algorithm implemented in Illumina GenomeStudio software was used to get the alleles from raw intensity data.

Genotyped data was quality controlled (QC) removing samples and markers using the following pipeline: exclusion of samples with ≥ 15% missing rates; exclusion of markers with non-called alleles; exclusion of markers with missing call rates > 0.05; exclusion of samples with ≥ 5% missing rates; exclusion of related samples (PI-HAT > 0.1875); exclusion of samples whose genotyped sex could not be determined; exclusion of samples with high heterozygosity rate (more than three times standard deviation (SD) from mean); only autosomal SNPs were kept; removal of markers with Hardy–Weinberg equilibrium P-value < 1 × 10^–5^; removal of markers whose P-value of difference in missingness between cases and control was < 1 × 10^–5^; and removal of samples which were outliers, identified using principal component analysis (deviation of more than 6 times interquartile range). At the end, 140 samples passed the QC.

Imputation of missing genotypes was done using TOPMed Imputation Server [23]. The reference panel used was TOPMed Version R2 on GRC38; and default pipeline was used. Once imputed, markers with INFO score < 0.80, MAF < 0.01 and non-biallelic markers were removed.

### Polygenic risk score

For PRS calculation, the results of a genome-wide association study about endometriosis [10] deposited in GWAS catalog with the accession number GCST004549 [24] was used as model. From the available SNPs, the 13 SNPs with *p*-value < 5 × 10^–8^ and present in our data were kept for the application of PRS.

Then, PRS was calculated as it is implemented in PLINK software [34] (version 1.9), using unweighted (presence of the risk allele) and weighted (using the beta value of the effect of the risk allele) methods.

### Principal components

Four principal components (PC) were calculated for each patient and used as covariates to control for population stratification. The genotyped data was pruned to get SNPs with no linkage disequilibrium using PLINK software [25] and SNPs from high LD regions were excluded. Then, FlashPCA was used to calculate the principal components of SNP data [26].

### Data categorization

Data was categorized as previously described [27].

### Statistical analyses

Statistical analyses were performed using the software SPSS^©^ statistical computer package version 26 for Windows. Values are presented as median and interquartile range or numbers and percentages. Since the quantitative data was not normally distributed, comparisons of basic characteristics between groups were performed by Mann–Whitney U test and PRS was divided into quartiles or the 10% with highest PRS compared with the rest. Fisher´s exact test was used for dichotomous variables and for comparison of disease distribution among quartiles. Logistic regression was used to calculate odds ratio (OR) with 95% confidence interval (CI) and p for trends with localization, symptom or hormonal treatment as dependent variable and weighted or unweighted PRS as independent variable, adjusted for PC1–PC4, using both lowest and highest quartiles for PRS as reference values. Since age differed between patients with isolated and spread endometriosis and between patients with and without hormone therapy, age was added as a covariate in these calculations. Receiver operating characteristic (ROC) curves were used to show area under the curve (AUC) with 95% CI for PRS. Generalized linear model was used to calculate β value and 95% CI with inflammatory proteins and TRAb as dependent variable and weighted or unweighted PRS and PC1-PC4 together with confounders as independent variables. *p* < 0.05 was considered statistically significant.

To investigate if combinations of proteins were associated with the PRS, a combination of cross-validation and LASSO-regression was employed. We divided the data into a training and test set using a randomizing split. The shrinkage parameter (λ) was estimated using k-fold cross-validation in the training set. To perform variable selection the estimated shrinkage parameter λ_CV_ was used in the test set. The selected variables and the absolute value of the coefficients were saved, and the process was repeated 10 times. Further, variables were ordered by the number of times they were selected and the sum of its estimated coefficients. The lowest ranked variable was removed, and the whole process was repeated until a final model was selected. This method has previously been described in Leandersson et al. (2020) [28]. The final models were estimated with linear regression and compared against an intercept only model with likelihood ratio (LR) tests. Cross-validation and LASSO-regression analyses were carried out using R v 4.0.0 [29].

## Results

### Subject characteristic

In total, 172 patients were included in the study (Table 1). Patients were divided into subgroups depending on isolated or spread endometriosis, with or without gastrointestinal involvement, gastrointestinal symptoms and hormone therapy (Additional file 3: Table S1–S4).

**Table 1:** Sociodemographic factors, lifestyle habits and gastrointestinal symptoms in endometriosis patients

Variables	Patients N = 172
**Age** (years)	38.0 (32.0–43.0)
**BMI**, kg/m^2^	24.3 (21.8–27.1)
**Current smoking**, n (%)	26 (15.1)
Missing value	1 (0.6)
**Alcohol intake ≥ 1 glass/week**, n (%)	64 (37.2)
**Education level**, n (%)	
Missing value	1 (0.6)
* Primary school*	5 (2.9)
* Secondary school*	31 (18.0)
* University or college degree*	135 (78.5)
**Occupation**, n (%)	
Missing value	1 (0.6)
* Working/student*	152 (88.4)
* Sick leave/unemployed*	19 (11.0)
**Physical activity ≥ 1 h/week**, n (%)	85 (49.4)
Missing value	1 (0.6)
**Marital status**, n (%)	
Missing value	6 (3.5)
* Single/Living alone*	51 (29.7)
* Married/cohabitation*	131 (65.7)
*Other*	2 (1.2)
**Current hormone therapy**, n (%)	80 (46.5)
**Opioids**, n (%)	30 (17.4)
**Prior abdominal surgery**, n (%)	165 (95.9)
**Visual analog scale for irritable bowel syndrome**	
Missing value	2 (1.2)
**Abdominal pain** (mm)	40 (9–72)
* Reference values*	5 (1–15)
**Constipation** (mm)	27 (0–69)
* Reference values*	9 (1–22)
**Diarrhea** (mm)	15 (0–55)
* Reference values*	3 (0–10)
**Bloating and flatulence** (mm)	55 (17–80)
* Reference values*	14 (1–29)
**Vomiting and nausea** (mm)	9 (0–63)
* Reference values*	2 (0–3)
**Psychological well-being** (mm)	30 (6–63)
* Reference values*	4 (0–16)
**Intestinal symptoms influence on daily life** (mm)	40 (7–75)
* Reference values*	2 (0–18)

Basic characteristics of patients with endometriosis. BMI = Body Mass Index. Values are presented as median (interquartile range) or numbers and percentages (%). Gastrointestinal symptoms were measured on the Visual Analog Scale for Irritable Bowel Syndrome, where 0 mm represents no symptoms and 100 mm represents very severe symptoms [20]. Reference values from healthy controls are shown [21].

Patients with isolated ovarian endometriosis had a higher age compared to patients with spread endometriosis (40.0 (34.0–45.0) years vs. 38.0 (31.0–42.0) years, *p* = 0.034) and fewer were physically active ≥ 1 h/week (37.3% vs. 57.0%, *p* = 0.012) (Additional file 3: Table S1). No differences in basal characteristics were observed between patinets with and without endometriosis involving the gastrointestinal tract (Additional file 3: Table S2). Patients without gastrointestinal symptoms had a higher age, (40.0 (34.0–46.5) years versus 38.0 (32.0–43.0) years, *p* = 0.036) and a lower prevalence of hormone therapy compared to patients with gastrointestinal symptoms (24.0% vs. 50.3%, *p* = 0.017) (Additional file 3: Table S3). Patients without hormone therapy had a higher age compared to patients with current hormone treatment (39.0 (33.0–44.0) years vs. 37.0 (31.0–42.0) years, *p* = 0.046) and were to a greater extent married or cohabitants than single or living alone, compared with patients with current hormone therapy (*p* = 0.002). There were some differences in gastrointestinal symptoms between patients with and without hormone therapy, with more abdominal pain, diarrhea and vomiting and nausea in those with hormone therapy (Additional file 3: Table S4). Smoking habits, alcohol habits, education, occupation, and opioid treatment were similar in the different subgroups (Additional file 3: Table 1–4).

### Genetical analyses

In the third quartile of weighted and unweighted PRS, a lesser number of patients had spread endometriosis compared to the lowest quartile (OR: 0.252; 95% CI 0.081–0.782, *p* = 0.017 and OR: 0.182; 95% CI 0.052–0.0630, *p* = 0.007, respectively) as well as compared to the highest quartile (OR: 0.409; 95% CI 0.136–1.288, *p* = 0.111 and OR: 0.245; 95% CI 0.077–0.781, *p* = 0.017). There was no significant association between the PRS and localization when calculated as p for trend (Table 2 and 3).

**Table 2:** Association of weighted PRS in different subgroups of endometriosis patients

			Crude OR (95% CI)	*p*-value	Adj. OR (95% CI)	*p*-value
Isolated/spread	Isolated/spread	*p*-value				
Quartile 1	10/23	0.111	1.000		1.000	
Quartile 2	15/18		0.522 (0.190–1.433)	0.207	0.496 (0.154–1.599)	0.240
Quartile 3	20/16		0.348 (0.129–0.938)	0.037	0.252 (0.081–0.782)	0.017
Quartile 4	11/23		0.909 (0.324–2.554)	0.857	0.616 (0.192–1.974)	0.415
P for trend						0.243
No GI tract involvement /GI tract involvement	No GI tract involvement /GI tract involvement	P-value				
Quartile 1	26/7	0.144	1.000		1.000	
Quartile 2	30/3		0.371 (0.087–1.581)	0.181	0.158 (0.026–0.949)	0.044
Quartile 3	28/8		1.101 (0.349–3.470)	0.870	0.950 (0.248–3.644)	0.940
Quartile 4	23/11		1.1776 (0.591–5.343)	0.306	1.962 (0.524–7.338)	0.317
P for trend						0.100
No GI symptoms/ GI symptoms	No GI-symptoms/ GI-symptoms	P-value				
Quartile 1	5/30	0.631	1.000		1.000	
Quartile 2	3/29		1.611 (0.352–7.364)	0.538	4.219 (0.509–34.978)	0.182
Quartile 3	7/29		0.690 (0.197–2.425)	0.563	0.317 (0.065–1.539)	0.154
Quartile 4	7/28		0.667 (0.190–2.345)	0.528	0.530 (0.102–2.761)	0.451
P for trend						0.138
No hormone therapy /hormone therapy	No hormone therapy /hormone therapy	P-value				
Quartile 1	18/17	0.902	1.000		1.000	
Quartile 2	18/16		0.941 (0.366–2.421)	0.900	1.052 (0.344–3.213)	0.930
Quartile 3	20/16		0.847 (0.333–2.155)	0.727	0.713 (0.243–2.087)	0.537
Quartile 4	21/14		0.706 (0.274–1.820)	0.471	0.613 (0.205–1.830)	0.380
P for trend						0.285

Adj = adjusted, CI = confidence, OR = odds ratio. PRS = polygenic risk scores. ORs and 95% CI were calculated using binary logistic regression model, adjusted for PC1, PC2, PC3 and PC4. The calculations with isolated/spread endometriosis, no GI-symptoms/GI-symptoms and no hormone/hormone therapy were also adjusted for age. The calculations with no GI-symptoms/GI-symptoms was also adjusted for hormone therapy. Fisher´s exact test was used to calculate the distribution of PRS quartiles among groups. P-values < 0.05 were considered statistically significant

**Table 3:** Association of unweighted PRS in different subgroups of endometriosis patients

			Crude OR (95% CI)	*p*-value	Adj. OR (95% CI)	*p*-value
Isolated/spread	Isolated/spread	*p*-value				
Quartile 1	8/19	0.027	1.000		1.000	
Quartile 2	17/23		0.570 0.202–1.607	0.288	0.459 (0.137–1.537)	0.206
Quartile 3	23/16		0.293 (0.103–0.832)	0.021	0.182 (0.052–0.630)	0.007
Quartile 4	8/22		1.158 0.364–3.680)	0.804	0.741 (0.197–2.781)	0.657
P for trend						0.349
No GI tract involvement /GI tract involvement	No GI tract not involvement /GI tract involvement	*p*-value				
Quartile 1	21/6	0.029	1.000		1.000	
Quartile 2	35/5		0.500 (0.136–1.843)	0.298	0.196 (0.037–1.044)	0.056
Quartile 3	33/6		0.656 (0.186–2.310)	0.512	0.413 (0.090–1.897)	0.256
Quartile 4	18/18		2.333 (0.728–7.479)	0.154	2.354 (0.557–9.950)	0.244
P for trend						0.065
No GI symptoms/ GI symptoms	No GI-symptoms/ GI-symptoms	*p*-value				
Quartile 1	5/24	0.238	1.000		1.000	
Quartile 2	3/36		2.500 (0.546–11.450)	0.238	5.515 (0.666–45.659)	0.113
Quartile 3	6/33		1.146 (0.313–4.196)	0.837	1.265 (0.238–6.722)	0.782
Quartile 4	8/23		0.599 (0.171–2.101)	0.424	0.410 (0.075–2.236)	0.303
P for trend						0.146
No hormone therapy /hormone therapy	No hormone therapy /hormone therapy	*p*-value				
Quartile 1	13/16	0.484	1.000		1.000	
Quartile 2	22/19		0.702 (0.270–1.824)	0.467	0.507 (0.161–1.600)	0.247
Quartile 3	25/14		0.455 (0.170–1.214)	0.116	0.250 (0.075–0.829)	0.023
Quartile 4	17/14		0.669 (0.242–1.852)	0.439	0.473 (0.139–1.604)	0.229
P for trend						0.128

Adj = adjusted, CI = confidence, OR = odds ratio. PRS = polygenic risk scores. ORs and 95% CI were calculated using binary logistic regression model, adjusted for PC1, PC2, PC3 and PC4. The calculations with isolated/spread endometriosis, no GI-symptoms/GI-symptoms and no hormone/hormone therapy were also adjusted for age. The calculations with no GI-symptoms/GI-symptoms was also adjusted for hormone therapy. Fisher´s exact test was used to calculate the distribution of PRS quartiles among groups. P-values < 0.05 were considered statistically significant

**Fig. 1:**
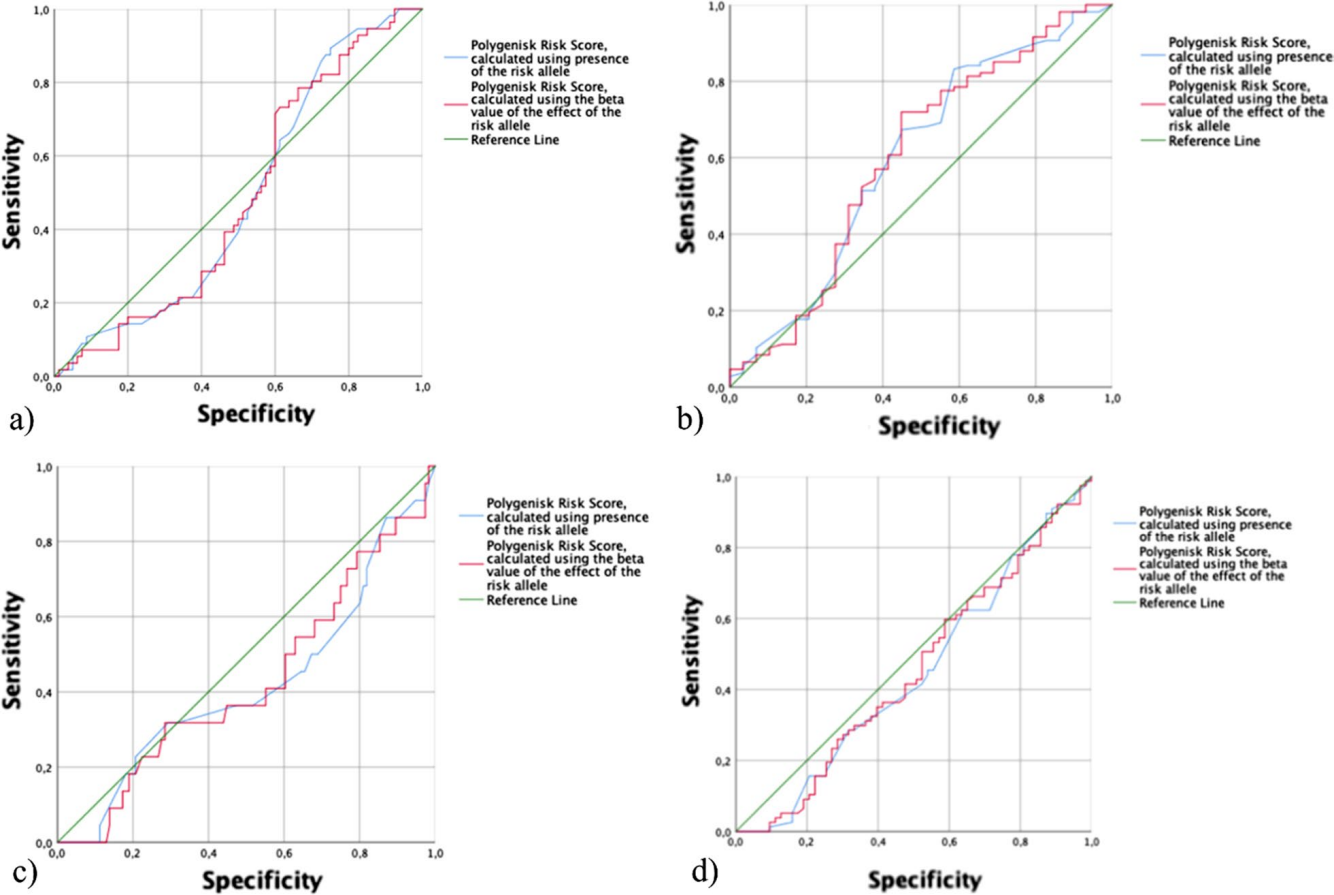
ROC-curves showing the sensitivity and specificity of PRS to predict isolated/spread endometriosis (**a**), GI tract not involved /GI tract involved (**b**), no GI symptoms/GI symptoms (**c**) and no hormone therapy /hormone therapy (**d**)

When calculating the associations between the 10% with highest PRS and localization of endometrios, no associations could be found (OR: 1.853; 95% CI 0.441–7.786; *p* = 0.400 for unweighted and OR: 1.886; 95% CI 0.428–8.311; *p* = 0.402 for weighted). The sensitivity and specificity of PRS to predict isolated or spread endometriosis was low (Fig. 1 a–d).

The second quartile of weighted PRS was associated with a lower risk of involvement of the gastrointestinal tract (OR: 0.158; 95% CI 0.026–0.949, *p* = 0.044), and the second quartile of unweighted PRS tended to be associated with a lower risk of gastrointestinal involvement (Table 2–3). The third quartile of unweighted PRS conferred with a lower risk of having hormone therapy (OR: 0.250; 95% CI 0.075–0.829, *p* = 0.023) (Table 3). There were no associations between PRS and TRAb or any of the inflammatory proteins investigated in this study (Table 4).

**Table 4:** Association of weighted and unweighted PRS, and inflammatory proteins and TSH receptor antibodies

	β (95% CI)	*p*-value
*Weighted PRS*		
AXIN1	− 1795.904 (− 4967.744 to 1375.936)	0.267
TRAb	− 2.496 (− 9.756 to 4.764)	0.500
ST1A1	7.033 (− 16.464 to 30.529)	0.557
CXCL9	14.556 (− 1.168 to 30.280)	0.070
OSM	7.276 (− 9.965 to 24.518)	0.408
MCP-1	1.713 (− 8.026 to 11.452)	0.730
TNFRSF9	4.951 (− 3.882 to 13.785)	0.272
*Unweighted PRS*		
AXIN1	− 190.936 (− 512.742 to 130.871)	0.245
TRAb	0.224 (− 0.760 to 1.209)	0.655
ST1A1	0.485 (− 1.896 to 2.384)	0.690
CXCL9	1.239 (− 0.358 to 2.837)	0.128
OSM	0.831 (− 0.908 to 2.570)	0.349
MCP-1	0.074 (− 0.910 to 1.059)	0.882
TNFRSF9	0.433 (− 0.461 to 1.327)	0.342

N = 140, β = beta coefficient, CI = confidence interval. Generalized linear model, adjusted for PC1, PC2, PC3 and PC4. P-values < 0.05 were considered statistically significant. Missing value AXIN1 = 4. Missing value TRAb = 7. Missing value ST1A1 = 64. Missing value CXCL9, OSM, MCP-1, TNFRSF9 = 62

**Fig. 2:**
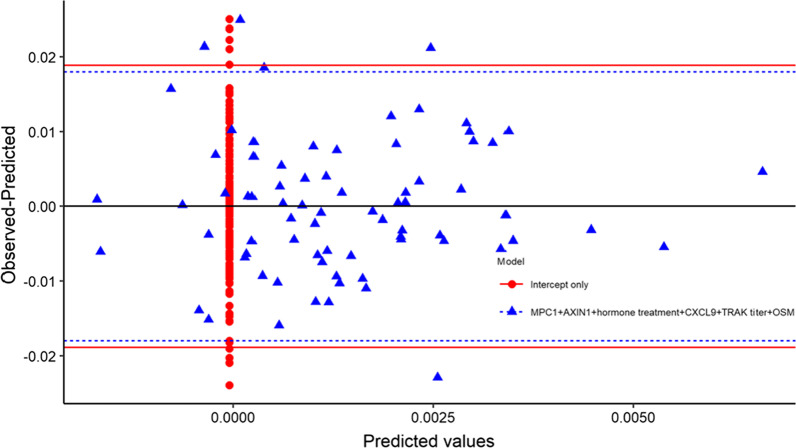
Residual plot from linear regression model for weighted PRS, comparing a model including MPC1, AXIN1, hormone therapy, CXCL9, TRAb titer, and OSM to an intercept only model. Red and blue horizontal lines are 0 ± 1.96*sd(residual)

**Fig. 3:**
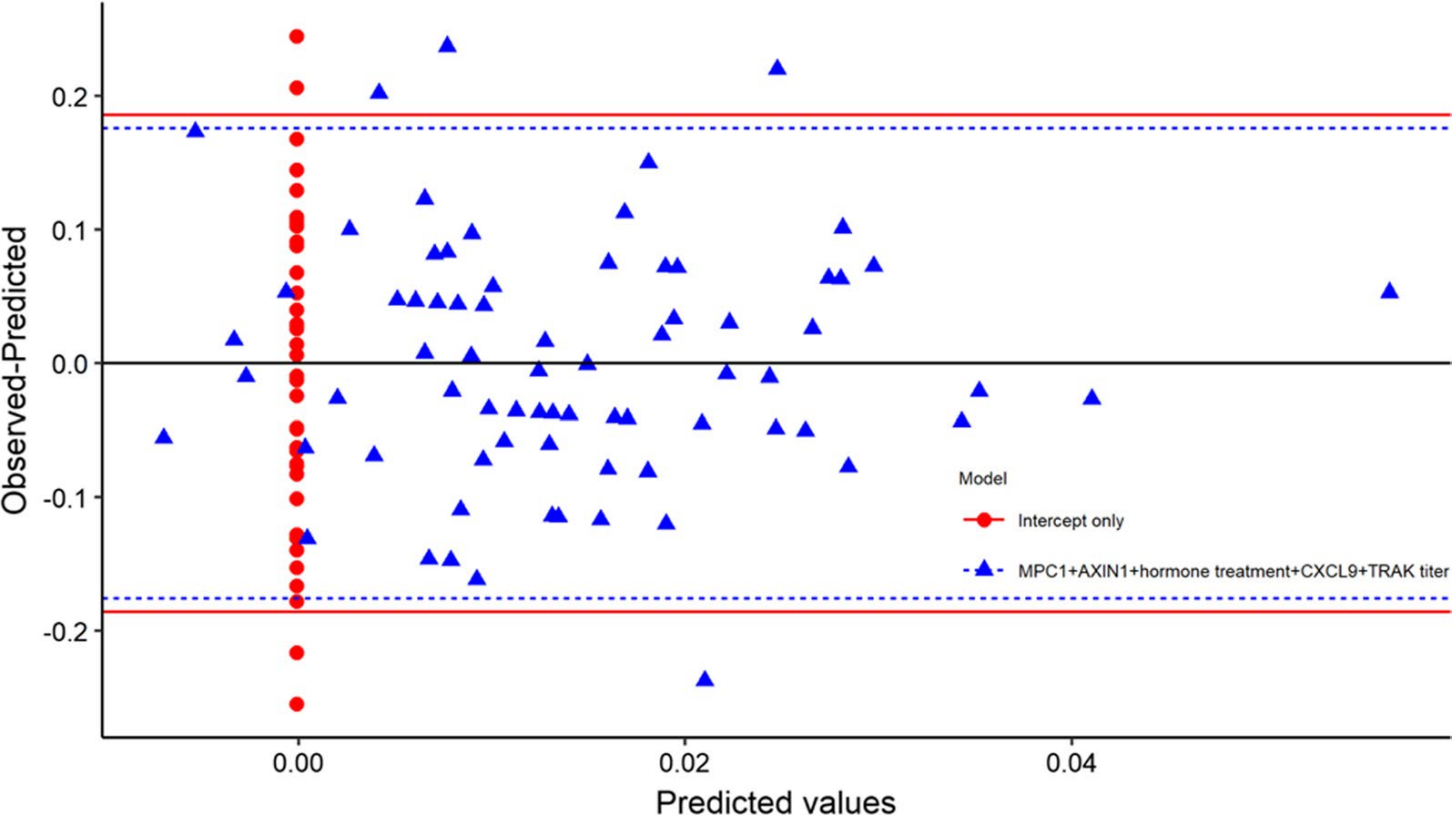
Residual plot from linear regression model for unweighted PRS, comparing a model including MPC1, AXIN1, hormone therapy, CXCL9, and TRAb titer to an intercept only model. Red and blue horizontal lines are 0 ± 1.96*sd(residual)

Calculations of combinations of proteins did not either show any associations between PRS and TRAb or inflammatory proteins (Figs. 2 and 3).

## Discussion

The main result in the present study was the inverse associations between the third quartile of weighted and unweighted PRS and spread endometriosis, between the second quartile of weighted PRS and gastrointestinal involvement, and the third quartile of unweighted PRS and hormone therapy. However, there seems to be no clinical use of PRS for endometriosis development to predict the clinical presentation in this entity since sensitivity and specificity for clinical outcome was low. There was no association between PRS and circulating inflammatory proteins or TRAb.

The clinical use of PRS has not yet been defined. For several diseases, PRS seems to be a promising clinical tool to calculate risks and treatment strategies, e.g., coronary artery diseases [30], dementia [31] and generalized epilepsy [32]. On the other hand, PRS is not enough to estimate the risk of coronary artery disease since non-genetic risk factors, such as age and environmental factors, are also involved [33]. When studying the association between PRS of low-density lipoprotein cholesterol (LDL-C) with circulating LDL-C levels and coronary artery disease, there were no associations within a cohort of patients with familial hypercholesterolemia [34]. Accordingly, PRSs are predictive of colorectal cancer in the general population but not in the Lynch syndrome [35]. Further, PRS was not able to have a great impact on discrimination models in some diseases, e.g., breast cancer [36].

Patients with endometriosis have an impaired psychological well-being compared with controls and are often diagnosed with depression and prescribed antidepressants [17]. Psychiatric comorbidity may exaggerate the experience of pain and gastrointestinal symptoms [37], however the causality in patients with endometriosis is unclear. We do not know whether endometriosis causes depression or whether depressed subjects have more severe symptoms and therefore more often seek health care and are admitted to a tertiary center. In a great community-based cohort, PRS for major depressive disorder, bipolar disease and schizophrenia were associated with all types of mental illness [38]. The comorbidity between depression and endometriosis may be a confounder interacting with SNPs found in GWAS and raise the question whether the associations found are representative for endometriosis or depression.

Our results show that genetic variants involved in developing endometriosis are not enough to explain the clinical presentation of the disease. For example, bowel symptoms, which are common in endometriosis patients, could be caused by other possible conditions such as microbiome dysbiosis [39], small bowel permeability [40] or perineal dysfunction [41]. Bowel symptoms could also be related to opioid treatment which is common in this group of patients [42] or caused by adherent tissue formation following abdominal surgery. Of the patients in our study 165 (95.9%) had undergone prior abdominal surgery. These factors affect the clinical presentation of endometriosis but are not possible to genetically identify. Endometriosis is known to be a chronic inflammatory disease and several studies have reported an increased level of inflammatory mediators in serum and peritoneal fluid [43, 44]. Further, the number of macrophages was higher in ovarian endometriomas tissue than in ovarian cysts [45]. SNPs associated with endometriosis in GWAS are encoding proteins involved in DNA-replication, transcriptional process, embryonic development, cytoskeleton regulation, angiogenic pathways, hormonal pathways and inflammatory pathways [10]. The interleukin 1A gene locus, involved in the regulation of proinflammatory cytokines and chemokine production, has been identified to have a significant association with endometriosis [43]. The associations found in GWAS of endometriosis thus implicate that SNPs involved in inflammatory regulation are associated with endometriosis. Taken together, the associations found in GWAS may represent depression and/or inflammatory conditions and are thus not representative for the clinical or laboratory outcome of endometriosis. These confounders may obscure the genetic picture.

GWAS of endometrios have identified genetic variants to differentiate endometriosis from healthy controls. The available genetic information is thus not intended to interrogate the genetic basis of the clinical presentation. Genetic variants involved in the risk of developing endometriosis seems not to be enough to explain the clinical or laboratory presentations of the disease.

Thus, it is needed to analyze the genetic variants involved in the disease presentation in a large cohort to develop useful PRS for this task. There are several limitations in this study. One of the most important limitations of this study is the small sample size of the cohort, recruited from a tertiary center. Another limitation is the limited characteristics without staging of the endometriosis cohort and no hormonal analyses.

## Conclusions

The genetic variants involved in the risk of developing endometriosis are not enough to explain the clinical presentations. Thus, the analysis of genetic variants involved in the clinical manifestations is needed to develop useful PRS for the clinical presentations.

## Supplementary Information

**Additional file 1:** Patient survey.

**Additional file 2:** Visual Analogue Scale for Irritable Bowel Syndrome (VAS-IBS).

**Additional file 3:** Supplementary Tables.

## Data Availability

Data available on request due to ethical restrictions.

## Abbreviations

AUC: Area under the curve
AXIN1: Axis inhibition protein 1
BMI: Body mass index
CXCL9: C-X-C motif chemokine ligand 9
ECLI: Electro chemi luminescence immunoassay
GI: Gastrointestinal
GnRH: Gonadotropin-releasing hormone
GWAS: Genome-wide association study
IBS: Inflammatory bowel disease
LR: Likelihood ratio
MCP-1: Monocyte chemoattractant protein 1
NPX: Normalized protein expression
OR: Odds ratio
OSM: Oncostatin-M
PC: Principal components
PEA: Proximity extension assay
PRS: Polygenic risk score
ROC: Receiver operating characteristic
SD: Standard deviation
SNP: Single nucleotide polymorphism
ST1A1: Sulfotransferase family 1A member 1
TNFRSF9: TNF receptor superfamily member 9
TSH: Thyroid stimulating hormone
TRAb: TSH receptor antibodies
QC: Quality controlled
VAS-IBS: Visual analog scale for irritable bowel syndrome
