# Supplementary material for: Applicability of polygenic risk scores in endometriosis clinical presentation
Source: BMC Womens Health. 2022 Jun 3;22:208. doi: 10.1186/s12905-022-01788-w (PMC9166598; doi:10.1186/s12905-022-01788-w)
Supplement: Supplementary file 1 — Additional file 1: Patient survey. [file 12905_2022_1788_MOESM1_ESM.doc]

# Patient survey UMAS

# Name………………………………………………………………................................

1. Social security number *(YYMMDD-XXXX)* _|_____|_____|_____|_____|_____|_____|_ – _|_____|_____|_____|_____|_

Civil status □1 living alone □ 2 married/partner

3. Check the option that best corresponds to the highest education you have received. Only one answer!

□ 1 Not completed primary school

□ 2 Primary school 1-9 years

□ 3 High school

□ 4 At least one year in addition to high school

□ 5 Degree from university or collage

1. Work (currently) □ Full time □Working 75 %

□ Working 50 % □ Working <50%

□ Sick leave □ Retired

□ Unemployed

1. How long have you been on sick leave due to your gastrointestinal symptoms in total over the years?

Number of months

4. Do you smoke? □ 1 Yes, smoke regularly

□ 2 Yes, smokes sometimes

□ 3 No, stopped smoking year ………

□ 4 No, have never smoked

1. If you have smoked, how many years have you smoked regularly, in total?____________________

5. Do you use snuff? □ 1 Yes

□ 2 No, stopped using snuff year_______

□ 3 No, have never used snuff

6. How many standard glasses (normal-sized glass for the intended beverage) do you drink on a normal week?_______________________

□1 Less than one standard glass/none

□2 1–4 standard glass

□3 5–9 standard glasses

□4 10–14 standard glasses

□5 15 or more standard glasses

8. How much time do you spend a regular week doing physical exercise that makes you breathless?

□1 No time at all

□2 Less than 30 minutes

□3 30–60 minutes (0,5−1 hour)

□4 60–90 minutes (1−1,5 hours)

□5 90–120 minutes (1,5−2 hours)

□6 More than 120 minutes (2 hours)

9. Weight today (kg) _|_____|_____|_____|_

10. Weight before problems started ( kg) _|_____|_____|_____|_

11. Length (cm) _|_____|_____|_____|_

12. What year did your stomach problems begin? _|_____|_____|_____|_____|_

13. What type of gastrointestinal symptoms has dominated? Rank by numbering the worst (1, 2, 3 …). If there is a problem that you do not suffer from, do not fill in the box.

□ 1 Abdominal pain □ 6 Vomiting
 □ 2 Diarrhea □ 7 Noise
 □ 3 Constipation □ 8 Bloating or gases
 □ 4 Nausea □ 9 Difficulty swallowing

□ 5 Heartburn, acid regurgitation

14. Have you had to change jobs due to your gastrointestinal symptoms?

□ 1 Yes □ 2 No

15. How did your gastrointestinal problems begin?

□ 1 Slow □ 2 Urgent/acute

16. Was there any special event that triggered your gastrointestinal symptoms?

□ 1 Yes □ 2 No

If yes, what?______________________________________________________________________

17. How have your gastrointestinal problems been over time?

□ 1 Constant □ 2 A little now and then □ 3 Gotten worse and worse

18. Do you have anyone in your family with similar problems?

□ 1 Yes □ 2 No

If yes, state number and family relationship

________________________________________________________________________________

________________________________________________________________________________

19. Have your gastrointestinal problems for any period required treatment with opioid or other pain-relieving treatment?

□ 1 Yes □ 2 No

If yes, for how long and which type?

_________________________________________________________

20. What name have your doctor given for your gastrointestinal problems?_____________________________________

21. What other diseases do you have besides your stomach problems?__________________________________________________________

22. What different examinations have you undergone for your stomach?

_____________________________________________________________________

Have you undergone in vitro fertilization?

□ Yes □ No

If yes, how many times? _|_____|_____|_

Which years? _|_____|_____|_____|_____| |_____|_____|_____|_____| |_____|_____|_____|_____| |_____|_____|_____|_____|_

16. If you are a woman, have you had hormone treatment due to gynecological chocolate cysts (endometriosis)?

□ 1 Yes □ 2 No

If so, state when and which type?______________________________________________

17. Have you received any other form of hormone treatment?

□ 1 Yes □ 2 No

If so, state when and which type?______________________________________________

18. Did any of your illnesses/ailments begin after undergoing hormone therapy or in vitro fertilization____________________________________________________________

19. Have you ever had surgery? □ 1 Yes □ 2 No

20. If so, state when and for what_____________________________________________

21. If applicable, enter the number

Pregnancies__________________

Vaginal births_________ delivery by cesarean section_______________________________

22. If you are a man, have you ever had hormone treatment for prostate cancer?

□ 1 Yes □ 2 No

If so, state when and which type?______________________________________________

23. Have you been treated for fallopian tube inflammation, chlamydia infection or gonorrhea?

□ 1 Yes □ 2 No

24. Have you been treated for herpes infection?

□ 1 Yes □ 2 No

25. List of current medications _________________________________________

_________________________________________

_________________________________________

_________________________________________

_________________________________________

26. Can you manage your nutrition with regular food?

□ 1 Yes □ 2 No

If no: □ 3 Need nutritional supplements

□ 4 Have tube feeding

□ 5 Have intravenous administration
