# Supplementary material for: Applicability of polygenic risk scores in endometriosis clinical presentation
Source: BMC Womens Health. 2022 Jun 3;22:208. doi: 10.1186/s12905-022-01788-w (PMC9166598; doi:10.1186/s12905-022-01788-w)
Supplement: Supplementary file 2 — Additional file 2: Visual Analogue Scale for Irritable Bowel Syndrome (VAS-IBS). [file 12905_2022_1788_MOESM2_ESM.doc]

Visual Analogue Scale for Irritable Bowel Syndrome (VAS-IBS)

VAS1. How have you been feeling during the past two weeks concerning abdominal pain?

Very good ___________________________________________ Very bad

VAS2. How have you been feeling during the past two weeks concerning diarrhea?

Very good ___________________________________________ Very bad

VAS3. How have you been feeling during the past two weeks in view of constipation?

Very good___________________________________________ Very bad

VAS4. How have you been feeling during the past two weeks concerning bloating and flatulence?

Very good ___________________________________________ Very bad

VAS5. How have you been feeling during the past two weeks concerning vomiting and nausea?

Very good ___________________________________________ Very bad

VAS6. How have you been feeling during the past two weeks concerning your psychological well-being?

Very good ___________________________________________ Very bad

VAS7. How much/little have your gastrointestinal problems influenced your daily life

over the past two weeks?

Not at all ___________________________________________ Very much

Have you during the past two weeks felt an urgency to defecate? 🞎 Yes 🞎 No

Have you during the past two weeks felt that your bowel has not been completely empty after visiting the toilet? 🞎 Yes 🞎 No
