# Supplementary material for: Applicability of polygenic risk scores in endometriosis clinical presentation
Source: BMC Womens Health. 2022 Jun 3;22:208. doi: 10.1186/s12905-022-01788-w (PMC9166598; doi:10.1186/s12905-022-01788-w)
Supplement: Supplementary file 3 — Additional file 3: Supplementary Tables. [file 12905_2022_1788_MOESM3_ESM.docx]

**Supplementary Tables**

**Supplementary Table 1.** Sociodemographic factors, lifestyle habits and gastrointestinal symptoms in patients with isolated ovarian endometriosis and where other locations are involved.

| **Variables** | **Isolated ovarian** N= 67 | **Spread** N= 100 | **P-value** |
| --- | --- | --- | --- |
| **Age**, years | 40.0 (34.0–45.0) | 38.0 (31.0–42.0) | 0.034 |
| **BMI**, kg/m^2^ | 24.3 (21.7–28.0) | 24.1 (21.8–26.8) | 0.646 |
| **Current smoking**, n (%) Missing value | 9 (13.4) | 17 (17.0) 1 (1.0) | 0.664 |
| **Alcohol intake ≥ 1 glass/week**, n (%) | 27 (40.3) | 36 (36.0) | 0.626 |
| **Education level**, n (%) Missing value | 1 (1.5) |  | 0.930 |
| *Primary school* | 3 (4.5) | 2 (2.0) |  |
| *Secondary school* | 12 (17.9) | 19 (19.0) |  |
| *University or college degree* | 0 (0.0) | 79 (79.0) |  |
| **Occupation,** n (%) Missing value |  | 1 (1.0) | 0.224 |
| *Working/student* | 60 (89.6) | 88 (88.0) |  |
| *Sick leave/unemployed* | 7 (10.4) | 11 (11.0) |  |
| **Physical activity ≥ 1 h/week**, n (%)  Missing value | 25 (37.3) | 57 (57.0) 1 (1.0) | 0.012 |
| **Marital status**, n (%)  Missing value | 2 (3.0) | 3 (3.0) | 0.915 |
| *Single/Living alone* | 19 (28.4) | 29 (29.0) |  |
| *Married/cohabitation* | 46 (68.7) | 66 (66.0) |  |
| *Other* | 0 (0.0) | 2 (2.0) |  |
| **Current hormone therapy**, n (%) | 27 (40.3) | 49 (49.0) | 0.342 |
| **Opioids**, n (%) | 9 (13.4) | 20 (20.0) | 0.304 |
| **Weighted polygenic risk score** Missing value | -0.00003161  (-0.020968–0.015760) 11 (16.4) | 0.00007617  (-0.023956–0.04995) 20 (20) | 0.738 |
| **Unweighted polygenic risk score** Missing value | -0.0008863  (-0.216771–0.167844) 11 (16.4) | 0.00213936  (-0.255233–0.244767) 20 (20) | 0.800 |
| **Visual analog scale for irritable bowel syndrome** |  |  |  |
| **Abdominal pain** (mm)  *Reference values* | 40 (7–75) 5 (1–15) | 40 (11–65) 5 (1–15) | 0.586 |
| **Constipation** (mm)  *Reference values* | 21 (0–70) 9 (1–22) | 32 (0–65) 9 (1–22) | 0.724 |
| **Diarrhea** (mm)  *Reference values* | 15 (0–60) 3 (0–10) | 10 (0–52) 3 (0–10) | 0.430 |
| **Bloating and flatulence**  *Reference values* | 60 (20–80) 14 (1–29) | 53.5 (12–76) 14 (1–29) | 0.487 |
| **Vomiting and nausea** (mm)  *Reference values* | 9 (0–50) 2 (0–3) | 8 (0–35) 2 (0–3) | 0.322 |
| **Psychological well-being** (mm)  *Reference values* | 32 (3–64) 4 (0–16) | 28.5 (10–59) 4 (0–16) | 0.842 |
| **Intestinal symptoms influence on daily life** (mm)  *Reference values* | 42.0 (7–75) 2 (0–18) | 40 (9–80) 2 (0–18) | 0.597 |

BMI = Body Mass Index. Gastrointestinal (GI) symptoms were measured on the Visual Analog Scale for Irritable Bowel Syndrome, where 0 mm represents no symptoms and 100 mm represents very severe symptoms [20]. Reference values from healthy controls are shown [21]. Values are presented as median (interquartile range) or numbers and percentages (%). The Mann-Whitney U or Fisher´s exact test. P-values <0.05 were considered statistically significant. Missing value = 5

**Supplementary Table 2.** Sociodemographic factors, lifestyle habits and gastrointestinal symptoms in patients with/without involvement of the gastrointestinal tract.

| **Variables** | **No GI involvement** N=134 | **GI involvement**  N=33 | **P-value** |
| --- | --- | --- | --- |
| **Age**, years | 38.0 (31.3–43) | 39.0 (34.5–43.0) | 0.211 |
| **BMI**, kg/m^2^ | 24.2 (21.8–27.1) | 24.7 (21.9–27.5) | 0.665 |
| **Current smoking**, n (%) Missing value | 20 (14.9) 1 (0.7) | 6 (18.2) | 0.604 |
| **Alcohol intake ≥ 1 glass/week**, n (%) | 52 (38.8) | 11 (33.3) | 0.689 |
| **Education level**, n (%) Missing value | 1 (0.7) |  | 0.285 |
| *Primary school* | 5 (3.7) | 0 (0.0) |  |
| *Secondary school* | 23 (17.2) | 8 (24.2) |  |
| *University or college degree* | 104 (77.6) | 25 (75.8) |  |
| **Occupation,** n (%) Missing value | 1 (0.7) |  | 0.965 |
| *Working/student* | 118 (89.4) | 29 (87.9) |  |
| *Sick leave/unemployed* | 14 (10.6) | 4 (12.1) |  |
| **Physical activity ≥ 1 h/week**, n (%)  Missing value | 64 (48.1) 1 (0.7) | 18 (54.1) | 0.563 |
| **Marital status**, n (%)  Missing value | 3 (2.3) | 1 (3.0) | 0.356 |
| *Single/Living alone* | 41 (30.8) | 7 (21.2) |  |
| *Married/cohabitation* | 87 (65.4) | 25 (75.8) |  |
| *Other* | 2 (1.5) | 0 (0.0) |  |
| **Current hormone therapy**, n (%) | 60 (45.1) | 16 (48.5) | 0.702 |
| **Opioids**, n (%) | 25 (18.8) | 4 (12.1) | 0.452 |
| **Weighted polygenic risk score** Missing value | -0.00075367  (-0.023956–0.023626) 27 (20.1) | 0.00292986  (-0.018008–0.00292986)  4 (12.1) | 0.099 |
| **Unweighted polygenic risk score** Missing value | -0.00636106  (-0.255233–0.244767)  27 (20.1) | 0.02766550  (-0.178310–0.244767) 4 (12.1) | 0.101 |
| **Visual analogue scale for irritable bowel syndrome**  Missing value | 2 (1.5) |  |  |
| **Abdominal pain** (mm)  *Reference values* | 45 (12–75) 5 (1–15) | 25 (4–67) 5 (1–15) | 0.195 |
| **Constipation** (mm)  *Reference values* | 32 (0–70) 9 (1–22) | 11 (0–58) 9 (1–22) | 0.175 |
| **Diarrhea** (mm)  *Reference values* | 12 (0–55) 3 (0–10) | 15 (0–55) 3 (0–10) | 0.936 |
| **Bloating and flatulence**  *Reference values* | 62 (20–80) 14 (1–29) | 31 (11–77) 14 (1–29) | 0.098 |
| **Vomiting and nausea** (mm)  *Reference values* | 11 (0–50) 2 (0–3) | 5 (0–28) 2 (0–3) | 0.435 |
| **Psychological well-being** (mm)  *Reference values* | 30 (11–65) 4 (0–16) | 20 (0–50) 4 (0–16) | 0.081 |
| **Intestinal symptoms influence on daily life** (mm)  *Reference values* | 45 (13–77) 2 (0–18) | 32 (3–65) 2 (0–18) | 0.183 |

BMI = Body Mass Index. Gastrointestinal (GI) symptoms were measured on the Visual Analog Scale for Irritable Bowel Syndrome, where 0 mm represents no symptoms and 100 mm represents very severe symptoms [20]. Reference values from healthy controls are shown [21]. Values are presented as median (interquartile range) or numbers and percentages (%). The Mann-Whitney U or Fisher´s exact test. P-values <0.05 were considered statistically significant. Missing value = 5

**Supplementary Table 3**. Sociodemographic factors, lifestyle habits and gastrointestinal symptoms in patients with gastrointestinal symptoms or not.

| **Variables** | **No GI symptoms** N=25 | **GI symptoms** N=145 | **P-value** |
| --- | --- | --- | --- |
| **Age**, years | 40.0 (34.0–46.5) | 38 (32–43) | 0.036 |
| **BMI**, kg/m^2^ | 24.2 (21.8–27.2) | 24.3 (21.8–27.1) | 0.925 |
| **Current smoking**, n (%) Missing value | 5 (20) | 21 (14.5) 1 (0.7) | 0.548 |
| **Alcohol intake ≥ 1 glass/week**, n (%) | 10 (40) | 53 (36.6) | 0.823 |
| **Education level**, n (%) Missing value |  | 1 (0.7) | 0.372 |
| *Primary school* | 1 (4.0) | 4 (2.1) |  |
| *Secondary school* | 3 (12.0) | 28 (19.3) |  |
| *University or college degree* | 21 (84.0) | 112 (77.2) |  |
| **Occupation,** n (%) Missing value | 1 (4.0) |  | 0.516 |
| *Working/student* | 24 (96.0) | 126 (86.9) |  |
| *Sick leave/unemployed* | 0 (0.0) | 19 (13.1) |  |
| **Physical activity ≥ 1 h/week**, n (%)  Missing value | 11 (44.0) | 73 (50.3) 1 (0.7) | 0.666 |
| **Marital status**, n (%)  Missing value |  | 5 (3.4) | 0.251 |
| *Single/Living alone* | 5 (20.0) | 46 (31.7) |  |
| *Married/cohabitation* | 20 (80.0) | 92 (63.4) |  |
| *Other* | 0 (0.0) | 2 (1.4) |  |
| **Current hormone therapy**, n (%) | 6 (24.0) | 73 (50.3) | 0.017 |
| **Opioids**, n (%) | 2 (8.0) | 27 (18.6) | 0.257 |
| **Weighted polygenic risk score**  Missing value | 0.002249200  (-0.011388–0.023626)  3 (12) | -0.00049760  (-0.023956–0.024995) 29 (20) | 0.268 |
| **Unweighted polygenic risk score**  Missing value | 0.02614655  (-0.128414–0.244767)  3 (12) | -0.00430588  (-0.255233–0.244767)  29 (20) | 0.229 |
| **Visual analogue scale for irritable bowel syndrome** |  |  |  |
| **Abdominal pain** (mm)  *Reference values* | 0 (0–0) 5 (1–15) | 50 (23–75) 5 (1–15) | 0.000 |
| **Constipation** (mm)  *Reference values* | 0 (0–0) 9 (1–22) | 41.5 (5.5–72)  9 (1–22) | 0.000 |
| **Diarrhea** (mm)  *Reference values* | 0 (0–0) 3 (0–10) | 20 (2–60) 3 (0–10) | 0.000 |
| **Bloating and flatulence**  *Reference values* | 0 (0–6) 14 (1–29) | 65 (32.5–82) 14 (1–29) | 0.000 |
| **Vomiting and nausea** (mm)  *Reference values* | 0 (0–0) 2 (0–3) | 15 (0–50) 2 (0–3) | 0.000 |
| **Psychological well-being** (mm)  *Reference values* | 0 (0–17) 4 (0–16) | 32 (15–69) 4 (0–16) | 0.000 |
| **Intestinal symptoms influence on daily life** (mm)  *Reference values* | 0 (0–0) 2 (0–18) | 51 (20–79) 2 (0–18) | 0.000 |

BMI = Body Mass Index. Gastrointestinal (GI) symptoms were measured on the Visual Analog Scale for Irritable Bowel Syndrome, where 0 mm represents no symptoms and 100 mm represents very severe symptoms [20]. Reference values from healthy controls are shown [21]. Values are presented as median (interquartile range) or numbers and percentages (%). The Mann-Whitney U or Fisher´s exact test. P-values <0.05 were considered statistically significant. Missing value = 2

**Supplementary Table 4.** Sociodemographic factors, lifestyle habits and gastrointestinal symptoms in patients with current/no current hormonal treatment.

| **Variables** | **No hormone therapy** N= 92 | **Hormone therapy** N= 80 | **P-value** |
| --- | --- | --- | --- |
| **Age**, years | 39.0 (33.0–44.0) | 37.0 (31.0–42.0) | 0.046 |
| **BMI**, kg/m^2^ | 24.1 (21.8–27.0) | 24.7 (21.8–27.4) | 0.607 |
| **Current smoking,** n (%)  Missing value | 13 (14.1) 1 (1.1) | 13 (16.3) | 0.832 |
| **Alcohol intake ≥ 1 glass/week,** n (%) | 32 (34.8) | 32 (40.0) | 0.529 |
| **Education level**, n (%) Missing value |  | 1 (1.3) | 0.966 |
| *Primary school* | 4 (4.4) | 1 (1.3) |  |
| *Secondary school* | 15 (16.3) | 16 (20.0) |  |
| *University or college degree* | 73 (79.3) | 62 (77.5) |  |
| **Occupation,** n (%) Missing value | 1 (1.1) |  | 0.138 |
| *Working/student* | 81 (88.0) | 71 (88.8) |  |
| *Sick leave/unemployed* | 10 (10.9) | 9 (11.3) |  |
| **Physical activity ≥ 1 h/week**, n (%)  Missing value | 51 (55.4) | 34 (42.5) 1 (1.3) | 0.126 |
| **Marital status**, n (%)  Missing value | 4 (4.4) | 1 (1.3) | 0.002 |
| *Single/Living alone* | 18 (19.6) | 33 (41.3) |  |
| *Married/cohabitation* | 68 (73.9) | 46 (57.5) |  |
| *Other* | 2 (2.2) | 0 (0.0) |  |
| **Opioids**, n (%) | 14 (15.2) | 16 (20.0) | 0.428 |
| **Weighted polygenic risk score**  Missing value | 0.00090289  (-0.15444–0.024995) 15 (16.3) | -0.00120544  (-0.023956–0.023729) 17 (21.3) | 0.335 |
| **Unweighted polygenic risk score** Missing value | 0.00955964 (-0.152753–0.244767)  15 (16.3) | -0.01186887  (-0.255233–0.244767) 17 (21.3) | 0.277 |
| **Visual analog scale for irritable bowel syndrome** Missing value |  | 1 (1.3) |  |
| **Abdominal pain** (mm)  *Reference values* | 35 (0–65) 5 (1–15) | 47 (14–80) 5 (1–15) | 0.033 |
| **Constipation** (mm)  *Reference values* | 25 (0–63) 9 (1–22) | 33 (0–70) 9 (1–22) | 0.425 |
| **Diarrhea** (mm)  *Reference values* | 10 (0–40) 3 (0–10) | 15 (3–63) 3 (0–10) | 0.031 |
| **Bloating and flatulence**  *Reference values* | 50 (10–80) 14 (1–29) | 62 (20–80) 14 (1–29) | 0.522 |
| **Vomiting and nausea** (mm)  *Reference values* | 4 (0–40) 2 (0–3) | 17 (0–50) 2 (0–3) | 0.048 |
| **Psychological well-being** (mm)  *Reference values* | 30 (10–53) 4 (0–16) | 35 (5–70) 4 (0–16) | 0.287 |
| **Intestinal symptoms influence on daily life** (mm)  *Reference values* | 35 (0–75) 2 (0–18) | 45 (15–78) 2 (0–18) | 0.189 |

BMI = Body Mass Index. Mm = millimeter. Gastrointestinal symptoms were measured on the Visual Analog Scale for Irritable Bowel Syndrome, where 0 mm represents no symptoms and 100 mm represents very severe symptoms [20]. Reference values from healthy controls are shown [21]. Values are presented as median (interquartile range) or numbers and percentages (%). The Mann-Whitney U or Fisher´s exact test. P-values <0.05 were considered statistically significant.
